# Description of changes in chemical bonding along the pathways of chemical reactions by deformation of the molecular electrostatic potential

**DOI:** 10.1007/s00894-024-06239-x

**Published:** 2025-01-03

**Authors:** Olga Żurowska, Artur Michalak

**Affiliations:** 1 Department of Theoretical Chemistry, Faculty of Chemistry, Jagiellonian University, Gronostajowa 2, 30-387 Krakow, Poland; 2 Doctoral School of Exact and Natural Sciences, Jagiellonian University, Łojasiewicza 11, 30-348 Kraków, Poland

**Keywords:** Deformation of molecular electrostatic potential, Deformation density, Chemical reactivity, Chemical bonding, Cycloaddition reaction, SN2 reaction, HCN/CNH isomerization assisted by water, CO + HF reaction

## Abstract

**Context:**

The analysis of the changes in the electronic structure along intrinsic reaction coordinate (IRC) paths for model reactions: (i) ethylene + butadiene cycloaddition, (ii) prototype SN2 reaction Cl^−^ + CH3Cl, (iii) HCN/CNH isomerization assisted by water, (iv) CO + HF → C(O)HF was performed, in terms of changes in the deformation density (Δr) and the deformation of MEP (ΔMEP). The main goal was to further examine the utility of the ΔMEP as a descriptor of chemical bonding, and to compare the pictures resulting from Δr and ΔMEP. Both approaches clearly show that the main changes in the electronic structure occur in the TS region. The ΔMEP picture is fully consistent with that based on Δρ for the reactions of the neutral species leading to the neutral products without large charge transfer between the fragments. In the case of reactions with large electron density displacements, the ΔMEP picture is dominated by charge transfer leading to more clear indication of charge shifts than the analysis of Δr.

**Methods:**

All the calculations were performed using the ADF package. The Becke–Perdew exchange–correlation functional was used with the Grimme’s dispersion correction (D3 version) with Becke-Johnson damping. The Slater TZP basis sets defined within the ADF program were applied. For the analysed reactions, the stationary points were determined and verified by frequency calculations, and the IRC was determined. Further analysis was performed for the structures of reactants, TS, products, and the points corresponding to the minimum and maximum of the reaction force. For each point, two fragments, A and B, corresponding to the reactants were considered. The deformation density was calculated as the difference between the electron density of the system AB and the sum of densities of A and B, $\Delta \rho \left(r\right)= {\rho }^{AB}\left(r\right)-{\rho }^{A}\left(r\right){-\rho }^{B}\left(r\right),$ with the same fragment definition as in the ETS-NOCV method. Correspondingly, deformation in MEP was determined as $\Delta V\left(r\right)={V}^{AB}\left(r\right)- {V}^{A}\left(r\right)- {V}^{B}\left(r\right)$.

## Introduction

In theoretical analysis of chemical reactions, it is common to utilize the concepts of the potential-energy profiles of chemical reactions on the Born–Oppenheimer potential-energy surface, *E*($\xi$), where $\xi$ represents the reaction coordinate. The most commonly used approach is based on *intrinsic reaction coordinate* (IRC) proposed by Fukui [1]. In analysis of the reaction mechanism, the importance and usefulness of the concepts of the reaction force [2] and the reaction force constant [3] was demonstrated in many examples [2–12]. The reaction force is defined as the first derivative of energy with respect to the reaction coordinate (negative energy gradient), and the reaction force constant as the corresponding second derivative (i.e., negative gradient of the reaction force). As a derivative of energy, the reaction force can be easily partitioned into contributions corresponding to the energy components discussed in energy decomposition analysis (EDA) methods. Politzer et al. [12] applied the energy-partitioning within the Activation Strain Model (ASM) proposed by Bickelhaupt [13–16] to discuss the reaction force components driving and retarding chemical reactions. In recent articles [17–19], we applied further decomposition of the interaction part of the reaction force, according to the Ziegler-Rauk energy decomposition scheme (extended transition state (ETS)) [20–22]. Decomposition of the reaction force into atomic contributions [23], and the related concepts of the reaction fragility spectra [24], and the connectivity matrix [25] were proposed in the groups of Komorowski and Ordon. Recently, the Symmetry-Adapted Perturbation Theory (SAPT) decomposition of the reaction force was presented by Derricote [26].

**Fig. 1:**
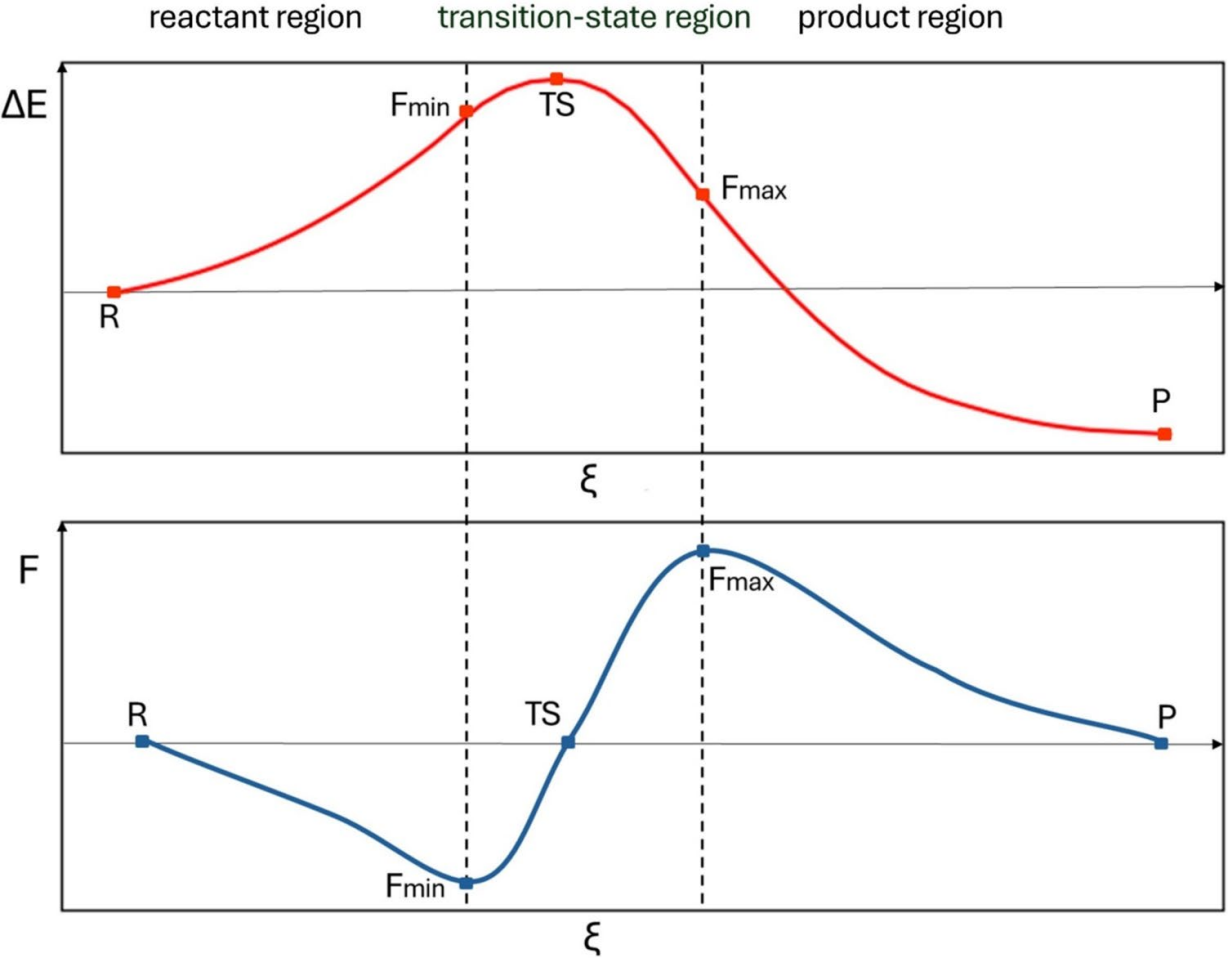
Example of typical IRC energy profile and the reaction force profile. The vertical lines divide the process into the reactant, transition state, and product regions. The characteristic points on the reaction pathway corresponding to reactant(s) (*R*), reaction force minimum (*F*_min_), transition state (TS), reaction force maximum (*F*_max_), and the product(s) (*P*) are indicated

The example IRC energy profile and the reaction force profile are schematically presented in Fig. 1. The reaction force vanishes for the structures of reactant(s) (R), transition state (TS), and the product(s) (P). Before the transition state, the reaction force is negative (retarding, i.e., acting against the reaction progress variable), and after TS it is positive (i.e., driving the system toward the product(s) the structure). Thus, it exhibits two extrema, minimum (for the structure corresponding to *F*_min_) and maximum (*F*_max_). These characteristic points of the reaction force provide a basis for the definition of distinctive regions of the reaction pathway: the reactant region (between *R* and *F*_min_), transition-state region (between *F*_min_ and *F*_max_) and the product region (between *F*_max_ and *P*). It was suggested that most of the electronic changes takes place in the TS region, while in the R and P regions, mostly the structural changes happen [6–8].

Molecular electrostatic potential (MEP) [27–30] *V*(**r**) represents energy of the electrostatic interaction of the molecular system with the unit, positive point charge located at point **r**, and as such, it contains nuclear and electronic contributions:

1 $${V}^{AB}\left(r\right)= \sum\nolimits_{j}\frac{{Z}_{j}}{|{R}_{j}-r|}- \int \frac{{\rho }^{AB}({r}{\prime})}{|{r}{\prime}-r|}{d}^{3}r{\prime}$$

In the above equation, *Z*_*j*_ represents the charge of nucleus *j* located at *R*_*j*_.

MEP is commonly used in a description of charge distribution in molecular systems, and in particular, as an important tool for diagnosing the chemical reactivity [27–30]. Due to primary importance of electrostatic interactions, the interpretation of MEP can also give important insight in a description of chemical bonding in molecular systems; among the most important examples one should mention the explanation of the nature of halogen bonding by MEP and the σ-hole concept [31–37]. In the context of chemical bonding, analysis of the MEP topology was also shown to be useful [38–40].

Recently, we proposed [41] to use deformation of MEP (differential MEP, ΔMEP), defined within a fragment-based approach, in a description of chemical bonding, as a tool supplementing the bonding analysis based on deformation density and the ETS-NOCV approach [42–44]. For two fragments (*A* and *B*), ΔMEP represents the difference between MEP of the molecular system and the sum of potentials of considered fragments (in the geometry of *AB*):

2 $$\Delta V\left(r\right)={V}^{AB}\left(r\right)- {V}^{A}\left(r\right)- {V}^{B}\left(r\right)$$

in analogy to the deformation density, $\Delta \rho \left(r\right)$, representing the difference

3 $$\Delta \rho \left(r\right)= {\rho }^{AB}\left(r\right)-{\rho }^{A}\left(r\right){-\rho }^{B}\left(r\right)$$

in the electron density of the molecular system *AB*, and the fragments *A* and *B.*

It is worth emphasizing that the deformation in MEP includes only the electronic part,

4 $$\Delta V\left(r\right)= - \int \frac{\Delta \rho ({r}{\prime})}{|{r}{\prime}-r|}{d}^{3}r{\prime}$$

since the atomic position are the same in the molecular system AB, and the considered fragments.

It can be expected from Eq. 4 that the regions with accumulation of electron density (positive $\Delta \rho$) in a molecule (compared to the fragments) should be primarily characterized by the negative ΔMEP, and the areas of electron density depletion (negative $\Delta \rho$) should be reflected by increase in ΔMEP (positive values). In this way, ΔMEP and $\Delta \rho$ pictures should contain similar information about formation of chemical bonds. However, MEP is energy-based quantity, and has long-range character. Thus, the value of ΔMEP calculated at a given point reflects changes in $\Delta \rho$ at all points in space (weighed by the inverse distance, Eq. 4). The examples presented in our previous paper [41] for various molecules indicate then ΔMEP is more sensitive than $\Delta \rho$ for large polarization of the fragments.

In our previous paper [41], we investigated ΔMEP, and compared the resulting picture with Δr, and its components from ETS-NOCV analysis for fundamental examples of N_2_ and ethane, followed by a series of organic molecules with different substituents, and examples of the systems with hydrogen bonding. In the previous article, ΔMEP was only used for the *equilibrium geometries*. The main goal of this article is to verify possible utility of ΔMEP in a description of *changes* in chemical bonding along the pathways of chemical reactions, by comparison with results of the analysis of deformation density, assuming the same definition of the considered fragments. The reactions chosen as examples include well-known model reactions: (i) ethylene + butadiene cycloaddition, (ii) prototype SN2 reaction Cl^−^ + CH_3_Cl, (iii) HCN→CNH isomerization assisted by water, (iv) HF + CO → C(O)HF. Two main criteria were used in selection of these reactions: (a) the bond formation/bond-breaking processes occurring along the reaction are known, intuitive, and well accepted; (b) they include different examples concerning the polarization of fragments and/or charge flow between the fragments. Concerning the latter criterion, in the cycloaddition reaction (i), the neutral reactants lead to the neutral products through neutral TS. In the SN2 reaction (ii), anionic and neutral reactants are involved and thus the charge transfer occurs according to the scheme A^(−)^ + B-A → A-B + A^(−)^. In the isomerization of HCN assisted by water (iii), a complex of neural reactants (HCN + H_2_O) leads to a complex of neutral products (CNH + H_2_O) through TS exhibiting an ion-pair character, (CN)^( −)^–-(H_3_O)^(+)^. Finally, reaction (iv) involves neural reactants and product, but with involvement of strongly polarized bonds. We would like to verify if ΔMEP correctly reflects changes in chemical bonding, but as well, whether it provides additional information compared to the deformation density.

### Computational details

All the calculations were performed using the Amsterdam Modeling Suite/Amsterdam Density Functional (AMS/ADF) package (version 2023.104) [45–47]. The Becke–Perdew exchange–correlation functional was used [48, 49], coupled with the Grimme’s dispersion correction (D3 version) [50, 51] with Becke-Johnson damping [52, 53]. The Slater TZP basis sets defined within the ADF program were applied. For each of the analyzed reactions, the stationary points were determined and verified by frequency calculations, the IRC was determined, and the points corresponding to the minimum and maximum of the reaction force were determined from the reaction force profiles obtained by numerical differentiation of the IRC energy profile. In the analysis of deformation density and deformation in MEP, performed for each reaction for the structures corresponding to the characteristic points (*R*, *F*_min_, TS, *F*_max_, *P*), two fragments, A and B, corresponding to the reactants were considered: butadiene and ethylene in (i), Cl^−^ and CH_3_Cl in (ii), HCN and H_2_O in (iii), and HF and CO in (iv). Thus, the deformation density was calculated as the difference between the electron density of the system AB and the sum of densities of A and B, $\Delta \rho \left(r\right)= {\rho }^{AB}\left(r\right)-{\rho }^{A}\left(r\right){-\rho }^{B}\left(r\right).$ Correspondingly, deformation in MEP was determined as $\Delta V\left(r\right)={V}^{AB}\left(r\right)- {V}^{A}\left(r\right)- {V}^{B}\left(r\right)$, with the same fragment definition as used in the fragment-oriented approach applied in the ADF program [45, 47], and in the ETS-NOCV method [44]. Namely, the deformation density is expressed in terms of orthonormal spin-orbitals of the fragments, obtained by separate Löwdin orthogonalizations of the occupied fragment orbitals and the virtual ones, followed by the Schmidt orthogonalization of the virtual set on the occupied set [44]$.$

## Results and discussion

**Fig. 2:**
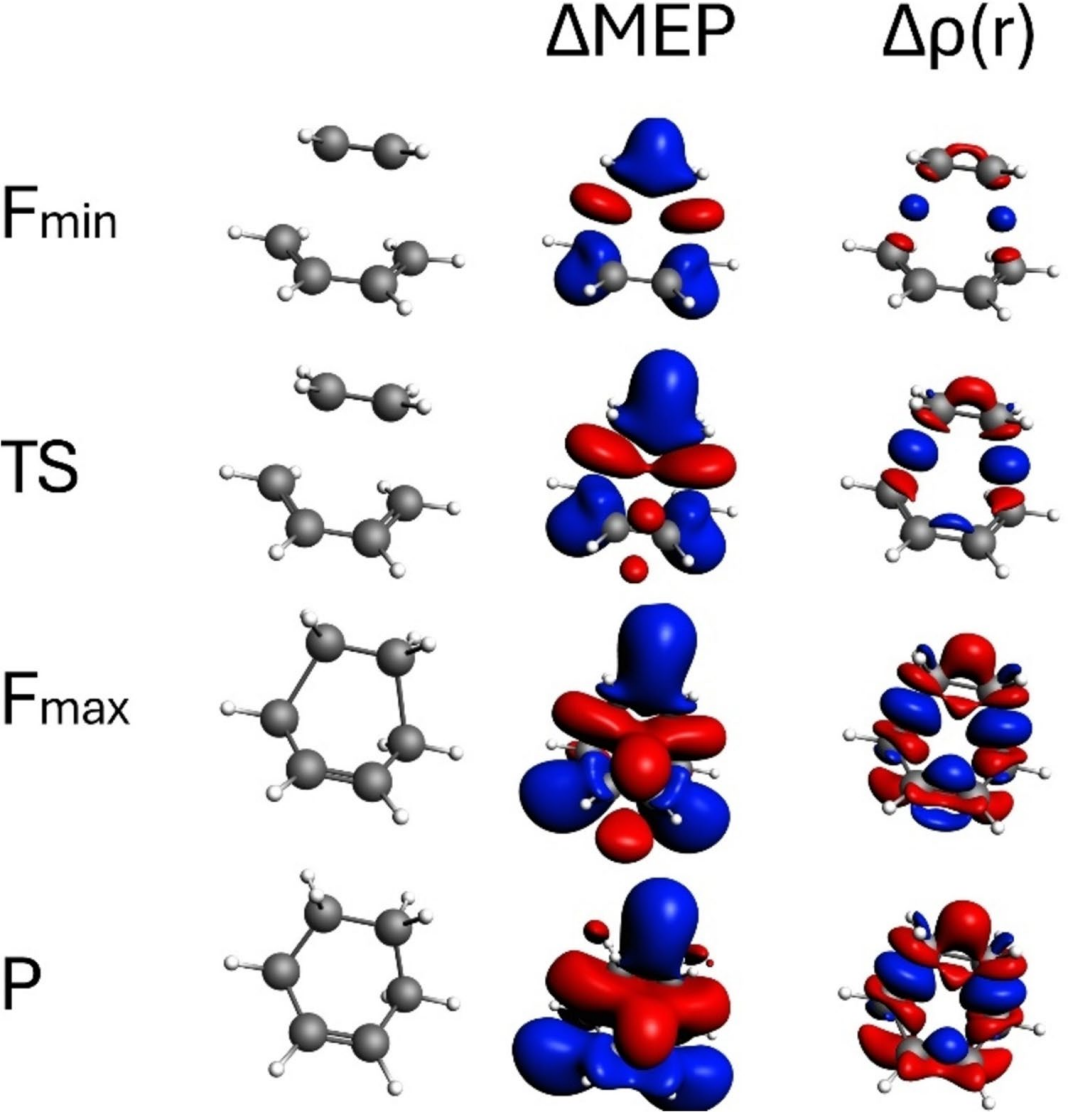
Contours of ΔMEP and Δρ along the reaction path for the Diels–Alder reaction of 1,3-butadiene and ethylene. The analysis is presented for characteristic points along the path: *F*_min_ (minimum reaction force), TS (transition state), *F*_max_ (maximum reaction force), and *P* (products). The contour value for ΔMEP is 0.01 a.u., and for Δρ, it is 0.005 a.u. Red and blue colour corresponds respectively to negative and positive values of ΔMEP/Δρ

**Fig. 3:**
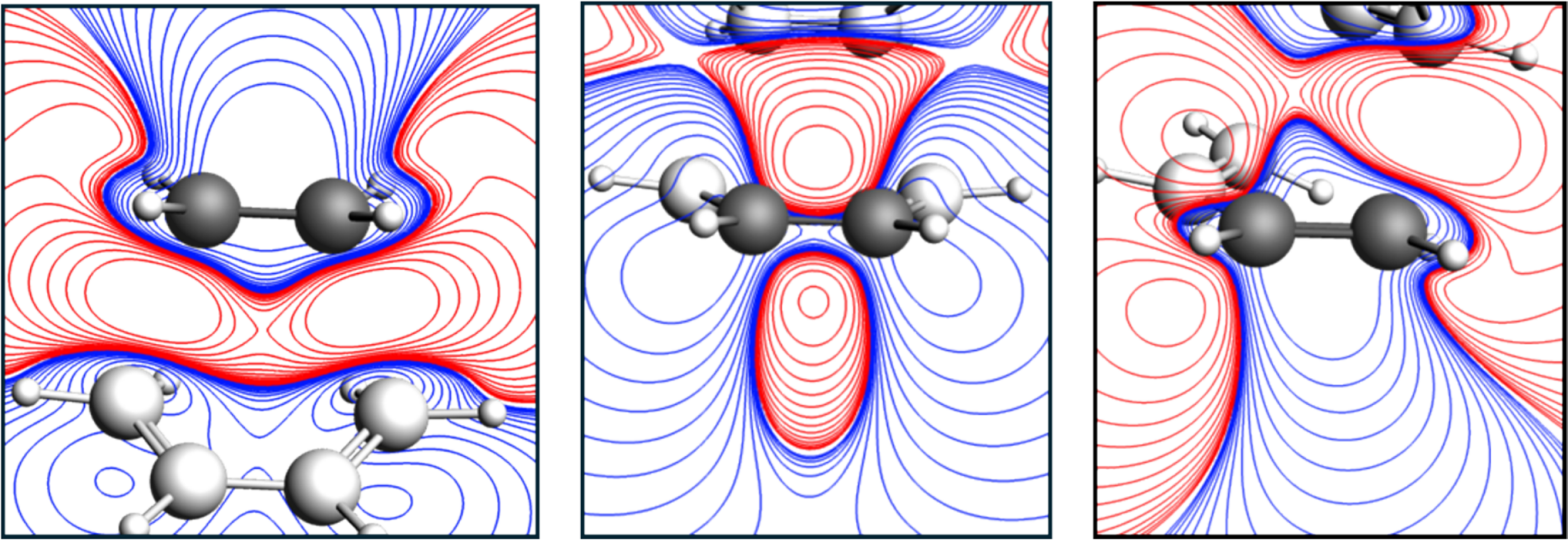
The contour maps showing cross-sections of ΔMEP for the structure of transition state for the Diels–Alder reaction of 1,3-butadiene and ethylene, plotted in the following planes: (left plot) containing the two ethylene carbon atoms; (middle plot) containing the two middle atoms of butadiene; (right plot) containing two terminal atoms of butadiene. For clarity, the atoms that are not in the plot plane are marked in white. Red and blue contours correspond to negative and positive values of ΔMEP, respectively

**Fig. 4:**
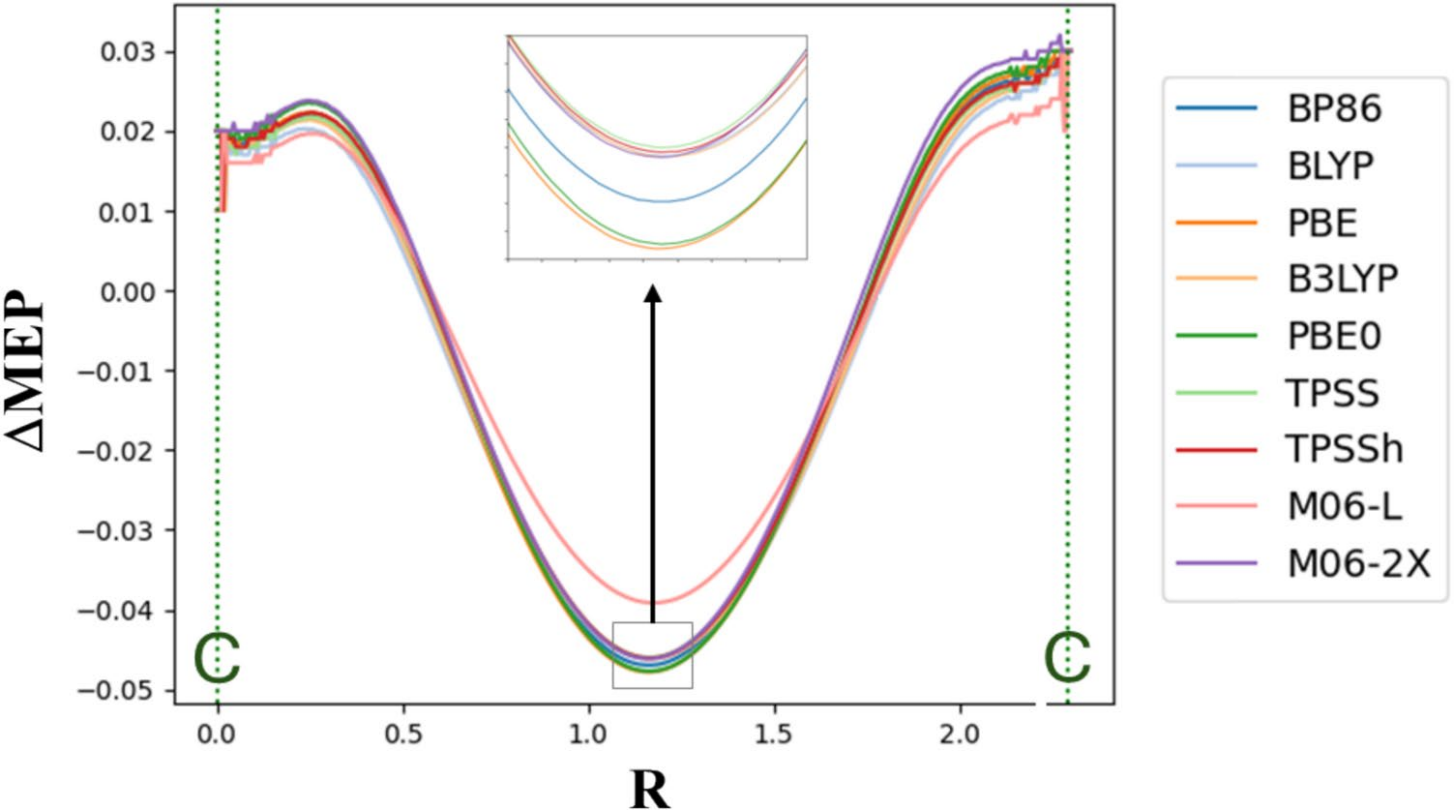
The ΔMEP values along the line connecting an ethylene carbon atom with a terminal carbon atom of butadiene for the structure of transition state for the Diels–Alder reaction of 1,3-butadiene and ethylene, calculated with various DFT exchange–correlation functionals (in a.u.)

**Fig. 5:**
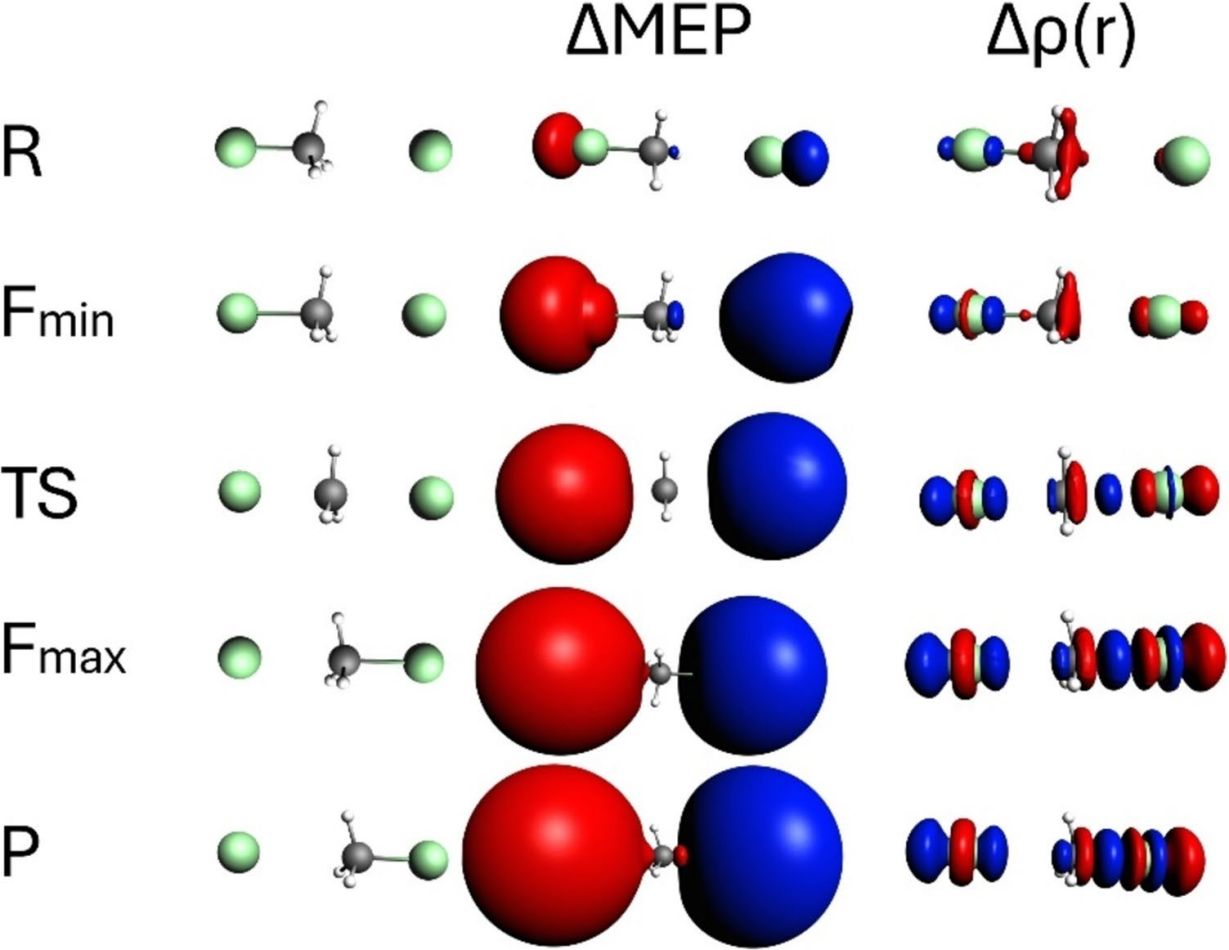
Contours of ΔMEP and Δρ along the reaction path for the SN2 reaction of CH_3_Cl with the Cl⁻ ion. The analysis is presented for characteristic points along the path: *R* (reactants), *F*_min_ (minimum reaction force), TS (transition state), *F*_max_ (maximum reaction force), and *P* (products). The contour value for ΔMEP is 0.04 a.u., and for Δρ, it is 0.005 a.u. Red and blue colour corresponds respectively to negative and positive values of ΔMEP/Δρ

**Fig. 6:**
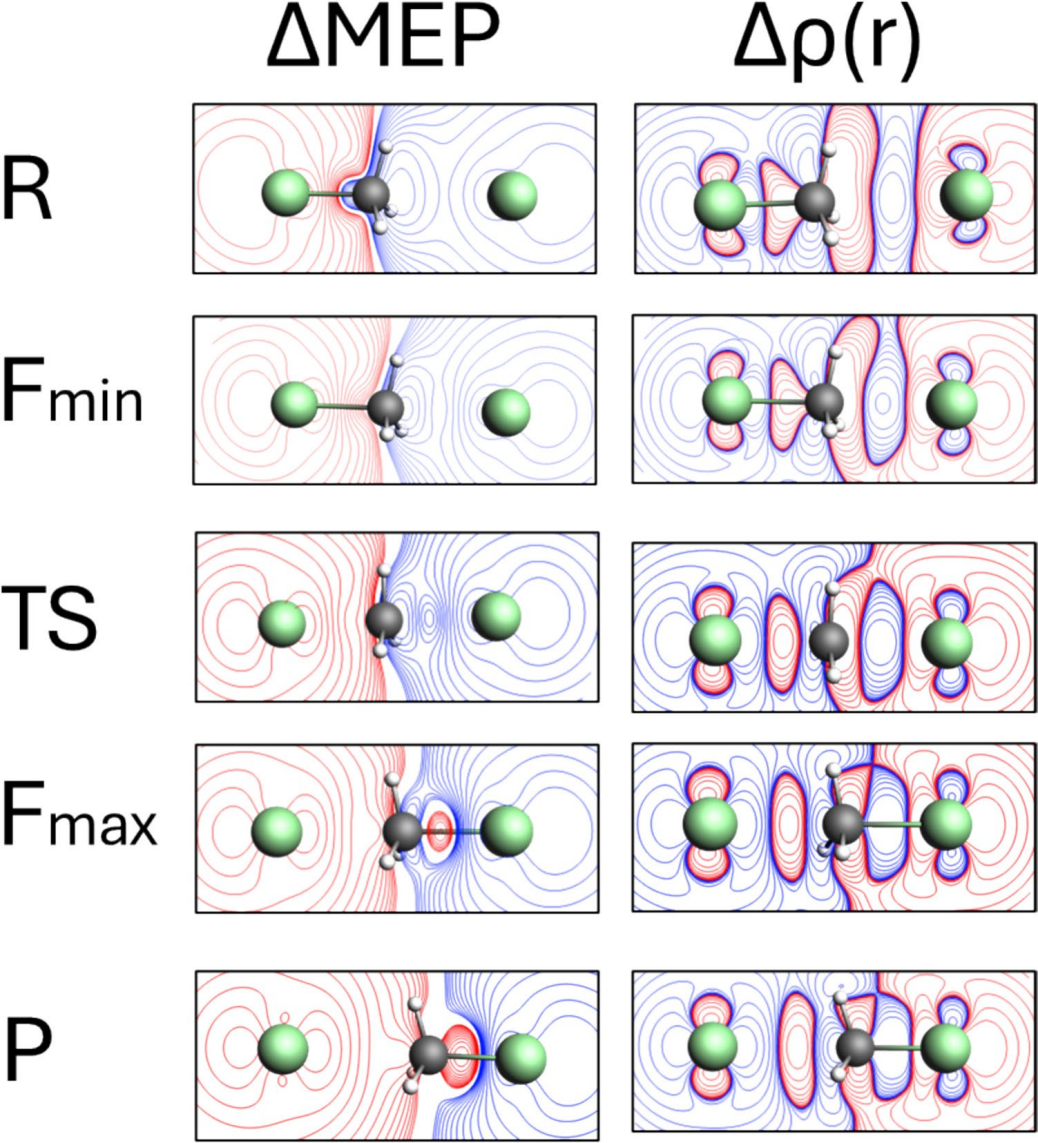
The contour maps showing cross-sections of ΔMEP and Δρ in the plane containing the two chlorine atoms and the carbon atom for the characteristic points along the reaction path for the SN2 reaction of CH_3_Cl with the Cl⁻ ion

**Fig. 7:**
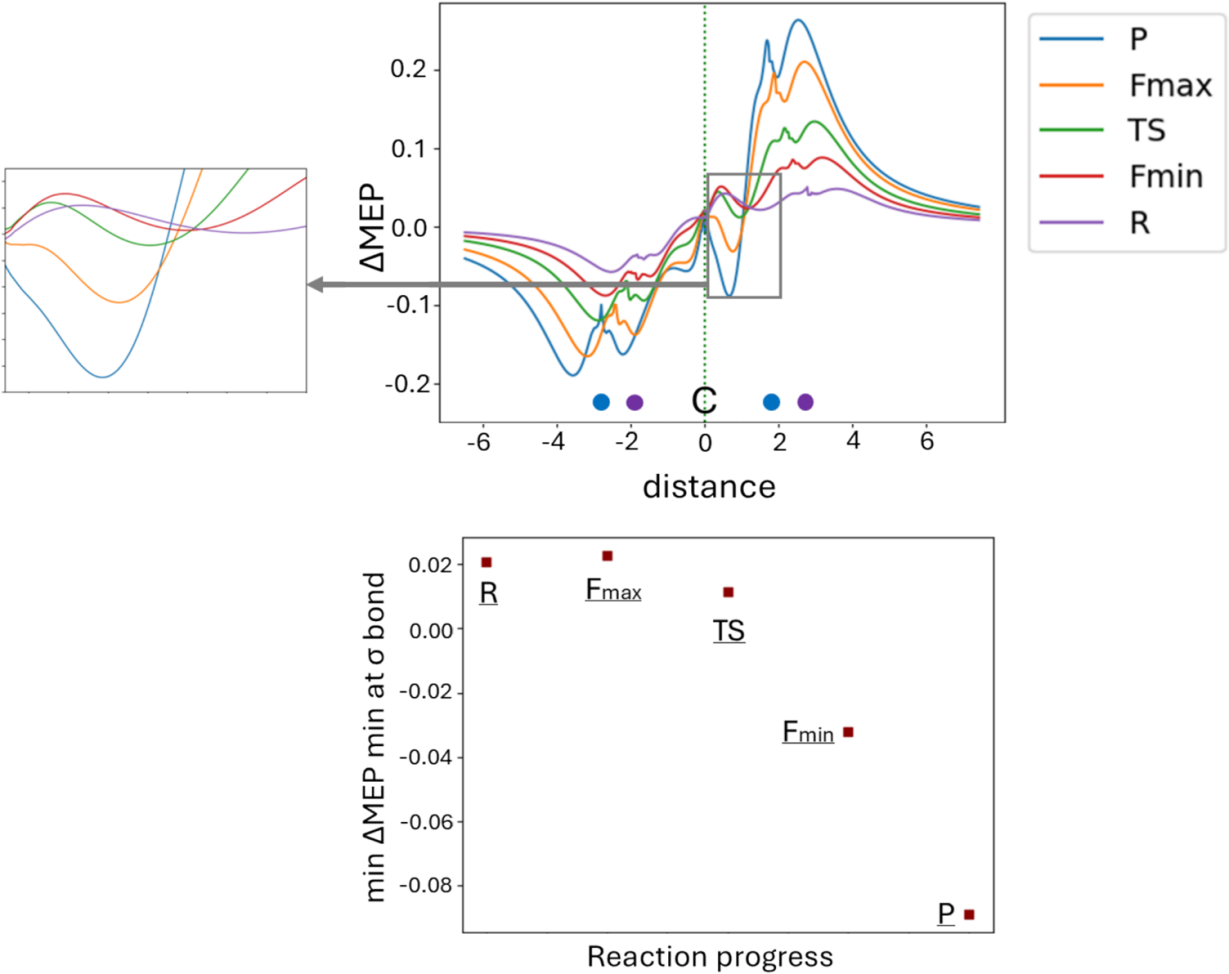
ΔMEP values along the line connecting the carbon atom with two chlorine atoms (top picture) for the geometries of *R*, *F*_min_, TS, *F*_max_, and *R*. The position of the carbon atom is marked by C (at 0.0 for all geometries), the position of the two chlorine atoms are marked by violet and blue circles, for *R* and *P*, respectively. The area of the C–Cl bond being formed during the reaction is magnified on the left-hand side of the main picture. The bottom picture presents the changes in the value of the ΔMEP minimum in this area along the reaction path

**Fig. 8:**
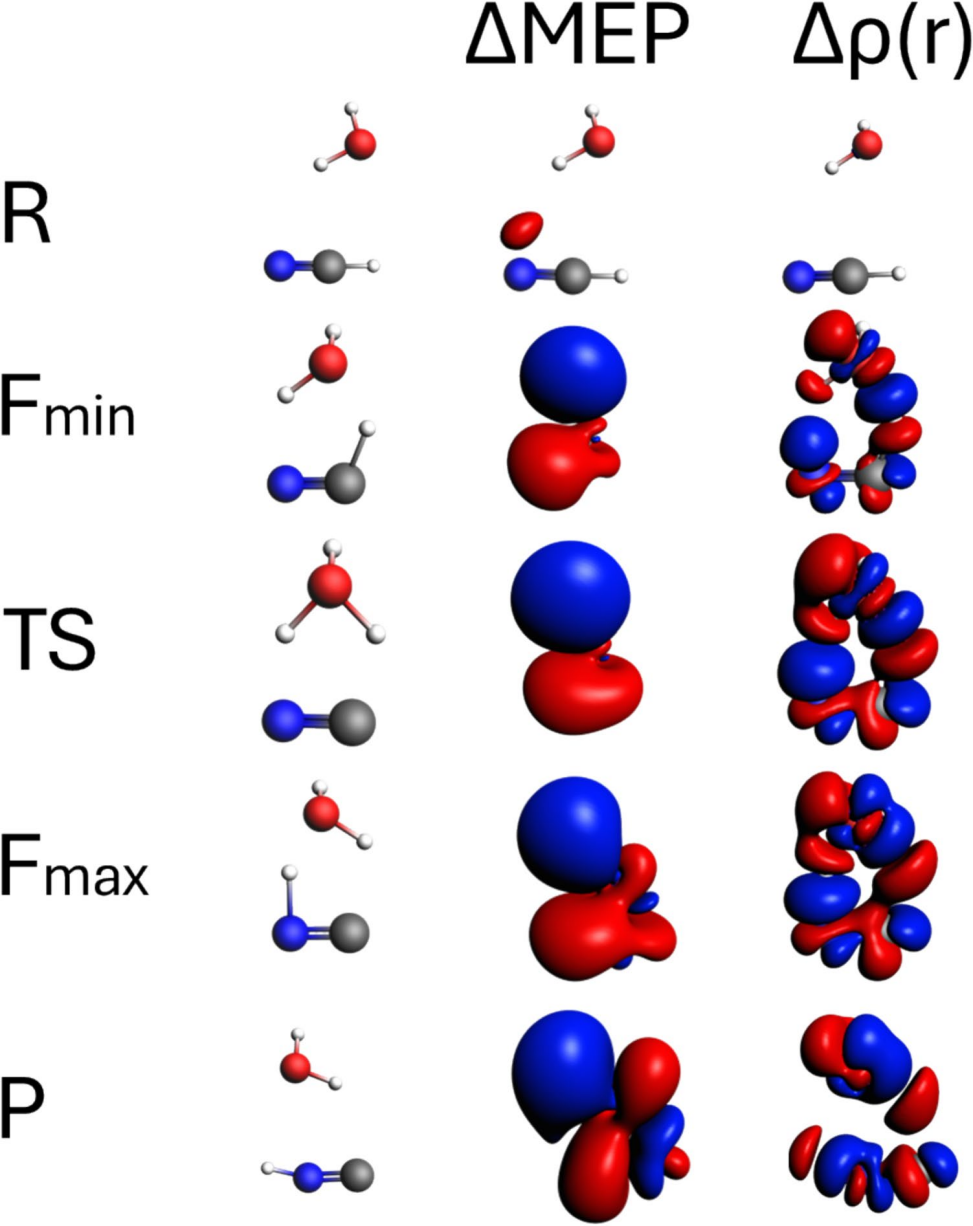
Contours of ΔMEP and Δρ along the reaction path HCN/CNH isomerization reaction assisted by water. The analysis is presented for characteristic points along the path: *R* (complex of reactants), *F*_min_ (minimum reaction force), TS (transition state), *F*_max_ (maximum reaction force), and *P* (products). The contour value for ΔMEP is 0.01 a.u., and for Δρ, it is 0.005 a.u. Red and blue colour corresponds respectively to negative and positive values of ΔMEP/Δρ

**Fig. 9:**
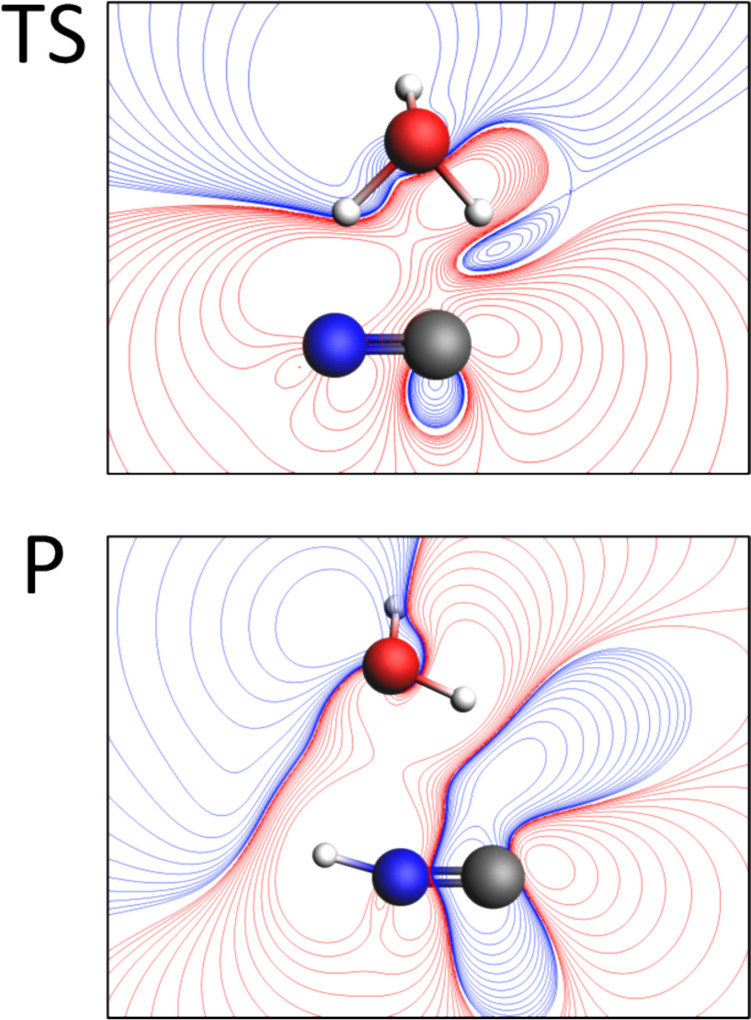
The contour map showing cross-section of ΔMEP for the structure of transition state (TS, top part) and the products (P, bottom part). Red and blue contours correspond to negative and positive values of ΔMEP, respectively

**Fig. 10:**
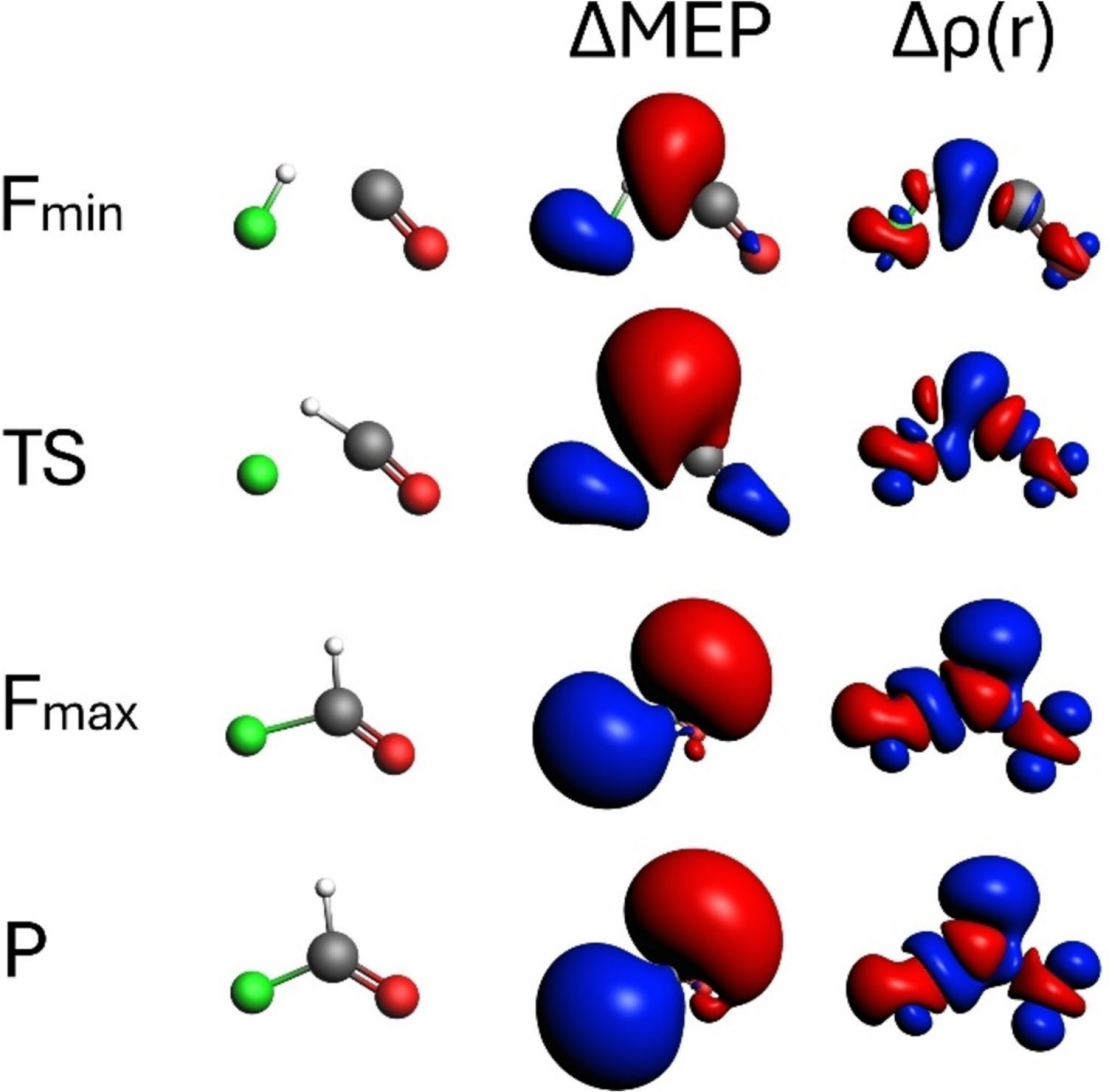
Contours of ΔMEP and Δρ along the CO + HF reaction path. The analysis is presented for characteristic points along the path: *F*_min_ (minimum reaction force), TS (transition state), *F*_max_ (maximum reaction force), and *P* (products). The contour value for ΔMEP is 0.02 a.u., and for Δρ, it is 0.005 a.u. Red and blue colour corresponds respectively to negative and positive values of ΔMEP/Δρ

In the following, for the reactions studied here, we will not present detailed analysis of the structures, the energetic features resulting from the IRC pathway, nor the reaction force profiles, since for these well-known reactions they have been discussed in the earlier papers. We will focus mostly on the analysis of the bond-formation and the bond-breaking processes with Δr and ΔMEP, for the reaction characteristic points (*R*, *F*_min_, TS, *F*_max_, *P*). For clarity, for each of the reactions discussed, the geometries are shown in Figs. 2, 3, 4, 5, 6, 7, 8, 9 and 10.

### The Diels–Alder cycloaddition reaction between 1,3-butadiene and ethylene

The mechanisms of Diels–Alder reactions was examined in numerous studies with use of a wide range of theoretical approaches [12, 54–62]. Cycloaddition reactions, particularly [4 + 2] processes, are now widely believed to proceed in a concerted fashion [54, 55, 58]. These reactions may exhibit varying levels of synchronicity, typically assessed via the reaction force constant, with the degree of synchronicity or nonsynchronicity often influenced by the specific structural features of the reactants [56, 58, 59]. In this study, we utilize the simple reaction of butadiene with ethylene, and we consider the concerted reaction pathway.

Contours of ΔMEP and Δρ along the reaction path for the Diels–Alder reaction of 1,3-butadiene and ethylene are shown in Fig. 2. The analysis is presented for characteristic points along the path: *F*_min_ (minimum reaction force), TS (transition state), *F*_max_ (maximum reaction force), and *P* (products); the van der Waals complex of interacting reactants is not included here because for this structure both ΔMEP and electron density difference are not visible for the given isocontour values. At *F*_min_, Δρ shows relatively minor changes, indicating initial electron density rearrangements. However, electron density accumulation (blue Δρ contour) in the areas between the carbon atoms of ethylene and the terminal carbon atoms of butadiene indicates initial stage of formation of covalent C–C bonds. This increase in electron density is accompanied by the decrease in the “atomic areas” of the carbon atoms of ethylene and the terminal carbon atoms of butadiene. These electron density changes are clearly reflected by the picture resulting from ΔMEP. Its exhibits negative values in inter-reactant region, in between the carbon atoms that form the bonds, and the positive values in the intra-reactant region. Positive ΔMEP values in the vicinity of the double C = C bonds (ethylene C = C, and the terminal C = C bonds of butadiene) indicates their initial weakening; one can see such changes in the p-electron region. It is worth pointing out here that these C–C bonds will become single bonds in the product (cyclobutene).

Approaching the transition state (TS), Δρ values visibly increase, highlighting substantial changes in electron density as new bonds are partly formed. The contours Δρ and ΔMEP corresponding to the changes in the electronic structure discussed above are magnified at TS compared to the *F*_min_-structure. The new features that were not yet visible at *F*_min_ include positive contour of Δρ in the area of the middle (“single”) C–C bond of butadiene, reflected by the negative ΔMEP contour in this area. Thus, at TS, one can see partial formation of the p-component of this C–C bond that will eventually become a double bond in the product.

In Fig. 3, the contour maps of ΔMEP are plotted, presenting the cross-sections for the TS structure in the planes containing C–C bonds of ethylene and butadiene. The contour-map representation of ΔMEP allows one to see more clearly the changes in the p-components of these bonds. In the case of ethylene, the extended area of the positive ΔMEP located above C–C bond corresponds to its weakening (double → single); the origin of the negative MEP (located in the same plot below the C–C bond) is the formation of the bonds between the carbon atoms of ethylene and the terminal carbon atoms of butadiene. In the case of butadiene, the contour-map representation clearly indicates formation of the π-component of the middle C–C bond (negative ΔMEP above and below this C–C bond), as well as decay of the π-components of the terminal C–C bonds (positive ΔMEP).

Contours of ΔMEP and Δρ (Fig. 2) at the *F*_max_-point more clearly (compared to TS) indicate (i) formation of the new C–C bonds (between the two reactants), (ii) double → single bond transition for the C–C bond in ethylene and the two terminal C–C bonds in butadiene; (iii) single → double bond transition for the middle butadiene C–C bond.

Finally, the contours of ΔMEP and Δρ for the product structure are practically indistinguishable from those at the *F*_max_-point. This indicates that all the bond-forming/bond-breaking processes happened in the transition-state region, and there are no major changes in the electronic structure in the product region, in agreement with the interpretation by Politzer and Toro-Labbe.

For the transition-state structure in the cycloaddition reaction, we performed additional calculations with use of various exchange–correlation functionals, to investigate their influence on ΔMEP. Figure 4 presents the ΔMEP values plotted along the line connecting the ethylene carbon atom with the terminal carbon atom of butadiene, obtained with a few popular exchange–correlation functionals of different classes. The results indicate their relatively minor influence on ΔMEP. In particular, the qualitative picture remains the same, and the variation in the specific values obtained with different functionals is by an order of magnitude smaller than the difference in ΔMEP minimum and maximum values along the line. The ΔMEP values obtained with BP86 functional, used in this study, are in between of the values calculated with other functionals. The largest deviation is observed for M06-L functional, not changing the qualitative picture, though.

### SN2 reaction of CH_3_Cl with the Cl⁻ ion

Several studies have explored the reaction mechanisms of SN2 processes and reaction force analysis, providing insights into nucleophilic substitutions in the gas phase, as well as into the solvent effects, along with advancements in understanding intrinsic reaction coordinates and reaction electronic flux [5, 63–72].

Starting with the changes in electron density difference Δρ (Fig. 5), at initial complex of reactants (R), their polarization is seen: a small decrease in electron density around the CH_3_ group and its accumulation in the area of the attached chlorine atom. At *F*_min_, Δρ shows the beginning of the C–Cl bond breaking (negative Δρ in the area corresponding to this bond), indicating initial electron density rearrangements as the Cl⁻ ion begins to approach the CH_3_Cl molecule. At the transition state (TS), Δρ reveals the formation of new C–Cl bond (positive Δρ between C and Cl), highlighting substantial changes in electron density as the nucleophile Cl⁻ ion forms a bond with the central carbon atom, while the leaving Cl atom starts to detach. At *F*_max_, Δρ retains its general shape but increases in magnitude. This corresponds to the highest electron density reorganization with clear evidence of bond formation/bond-breaking processes. Finally, at the product stage (P), Δρ looks very similar to that at *F*_max_, indicating that mostly relaxation in geometry occurs in the product region, without substantial changes in the electronic structure.

In the case of the ΔMEP contours, the picture looks quite different: the deformation in MEP clearly illustrates the charge transfer effect. As we indicated in the Introduction, here we have a reaction of neutral molecule + anion (Cl–CH_3_ + Cl^−^) giving anion + neutral molecule (Cl^−^ + CH_3_–Cl). A long-range character of molecular electrostatic potential implies that the large charge-flow between the fragments determines the overall picture: large red areas (negative ΔMEP) are clearly visible near the departing chlorine atom (initially “neutral,” becoming anion), and the blue areas (positive ΔMEP) around the chlorine (initially anion—becoming “neutral”) forming a new bond with the central carbon. The disparity in the magnitude of observed effects, i.e., charge transfer vs. bond formation makes the latter being practically invisible in the ΔMEP contours. This example nicely shows that ΔMEP analysis may serve as valuable tool, complementary do deformation density. Please note that in the sequence of the Δρ plots (*R* →* F*_min_ → TS → *F*_max_ →* P*), many Δρ changes accompanying bond formation/bond breaking makes the large charge transfer hidden. In the ΔMEP picture, it is opposite: the charge transfer is clearly seen, while the changes due to the local bond formation/bond-breaking processes are hidden “inside” the largest contours of ΔMEP.

In Figs. 6 and 7, two other graphical representations of ΔMEP are presented, to more deeply analyze the picture of bond-breaking, and bond formation processes, and charge transfer/polarization of fragments. Namely, in Fig. 6, the contour maps of ΔMEP and Δr, plotted in the plane containing the carbon atom and the chlorine atoms, are compared. In Fig. 7, the ΔMEP values along the line connecting the carbon atom with two chlorine atoms are plotted for all the considered geometries. In the contour maps of both deformation density and deformation in MEP, the initial polarization of reactants and some charge flow between them is seen already at *R* and *F*_min_. At TS and *F*_max_, presence of the deep minimum and maximum of Δr between the carbon atom and the chlorine atoms clearly indicates a Cl–C bond breaking, and formation of the other C–Cl bond. As in the case of the contour representation, the ΔMEP picture is dominated by the charge flow between the fragments. However, the C–Cl bond formation is reflected by the minimum of ΔMEP between the carbon and chlorine atom. This minimum is even more clearly seen in the linear representation of ΔMEP in Fig. 7. In the geometries corresponding to *F*_min_ and TS, the ΔMEP value at this minimum is still positive due to charge transfer (already relatively large), At *F*_max_ and *P*, the value at this minimum becomes negative, reflecting large increase in electron density due to the bond formation. It is more difficult to detect the Cl–C bond breaking in the ΔMEP plots, again—due to charge transfer. However, in the contour maps of Fig. 6, the changes in shape of the red (negative) ΔMEP contours in the vicinity of the Cl atom (becoming anionic) indicate a transition of sσ-symmetry orbital into the *p* orbital of Cl^−^, when going from *F*_min_ to *P*.

### HCN/CNH isomerization reaction assisted by water

The isomerization reaction between HCN and CNH has been extensively studied across various theoretical frameworks [57, 63, 64, 68, 73, 74], including reaction force analysis to provide insight into electronic shifts and mechanistic details. Water-assisted isomerization has also been investigated using ab initio [75] and DFT methods [17, 76–78]. It is known from these studies that the participation of water molecule(s) in the mechanism is important for lowering the activation barrier of the isomerization. For a pathway involving a single water molecule, reaction force and ETS-NOCV analyses were applied [17]. In the context of reactions on icy grain surfaces in the interstellar medium, pathways involving one to four water molecules were considered [75–78]. Recent studies suggest that the proton relay mechanism explicitly requires four water molecules, while additional, more distant water molecules are required for fine-tuning the process [77]. For a pathway involving a single water molecule, reaction force and ETS-NOCV analyses were applied [17]. In this article, the analysis of ΔMEP will be performed for this pathway with a participation of single water molecule [17].

The water-assisted isomerization reaction may serve, in a sense, as an intermediate case between the two reactions discussed above. Along the pathway of the HCN/CNH isomerization assisted by water, the hydrogen atom (or proton) from HCN is transferred to water and another hydrogen atom from water is transferred to form a bond with the nitrogen atom of CN group. Thus, the transition state can be considered an ion-pair H_3_O^+^CN^−^, and the mechanism of this reaction may be describe in a simplified form as $\mathrm {neutral\;molecule\:+\:neutral\;molecule\;\rightarrow\;\lbrack ion\;pair\rbrack\;\rightarrow\;neutral\;molecule\:+\:neutral\;molecule}$.

The Δρ contours shown in Fig. 8 indicate the gradual formation of the H–N bond between the hydrogen atom from the water molecule and the nitrogen atom, accompanied by the breaking of the C–H bond (of HCN), and the O–H bond. These changes in the electronic structure start to be visible at *F*_min_. When going from *F*_min_, through TS, to *F*_max_, the magnitude of the Δρ changes corresponding to the aforementioned bond breaking/bond processes increases. No further bond changes are visible at *P*. The bond formation and bond breaking were practically finalized at *F*_max_.

Concerning ΔMEP picture, in the complex of isolated reactants, only small polarization may be detected. The transition state exhibits significant charge transfer, which is highly visible in the ΔMEP representation (and not that clear in Δρ, hard to detect because of many negative/positive changes in different areas) already at *F*_min_. Thus, similar to the SN2 reaction, the overall ΔMEP picture is dominated by large charge shifts, especially when the isocontour representation is used. However, some details corresponding to specific bond formation/bond-breaking processes may be clearly visible in different graphical representations of the deformation in MEP. As an example, in Fig. 9, the contour maps corresponding to the cross-section of ΔMEP for the structure of transition state (TS, top part) and the products (P, bottom part) are shown. At TS, the negative areas of ΔMEP clearly indicate the partial formation of O–H and N–H bonds.

### The CO + HF reaction

The HF + CO → C(O)HF reaction, though less extensively studied, has been investigated [23–25, 79–81], focusing on conceptual DFT analysis, atomic resolution for the energy derivatives, and reaction fragility along the reaction path.

The reaction of CO with HF is an example of another addition reaction between two neutral molecules leading to the neutral product molecule. However, the CO + HF reaction is quite different from the cycloaddition reaction discussed in the beginning, since the large electronegativity of the fluorine atom results in quite substantial “local” charge displacements. The results of the Δρ and ΔMEP analysis are shown in Fig. 10. The Δρ changes along the reaction path demonstrate the progressive formation of new H–C and C–F bonds, alongside the breaking of the H–F bond. The changes in electron density are also observed in the C = O bond area, with electron density shift towards the oxygen atom. At *F*_min_, the initial bond formations and electron density adjustments are evident. At TS, Δρ highlights significant bond formation and substantial electron density rearrangement, especially in regions where bonds are transitioning. By *F*_max_, the electron density is maximized around the newly formed bonds. Electronic structure of the product is very similar to that at *F*_max_, as practically no changes are visible in the Δρ contours.

In ΔMEP plots, large charge displacements are evident. The most substantial electronic structure reorganization occurs between the TS and *F*_max_, with notable charge redistribution. The TS shows significant changes, while *F*_max_ reveals the peak of the electronic reorganization. At the product stage (P), ΔMEP picture is quite similar to *F*_max_, which is consistent with the Δρ picture. Thus, similar to the previous reaction discussed, in the product region, mostly the relaxation of geometry occurs, without substantial changes in the electronic structure. This is a slightly different picture from the results of Komorowski et al. for this reaction, based on the reaction fragility spectra [24, 73], suggesting that C–F bond formation occurs mostly in the final part of the reaction. Our results clearly show that the accumulation of electron density between HF and C appears already at *F*_min_, and at TS, the deformation density contour clearly shows formation of both H–C and F–C bonds, which is also reflected by negative contour of ΔMEP.

## Concluding remarks

In the present work, the analysis of the changes in the electronic structure along IRC paths for example, model reactions were presented, in terms of changes in the deformation density and the deformation in MEP. The main goal was to further examine the utility of the latter as a descriptor of chemical bonding. For the examined reactions, both approaches clearly show that the main changes in the electronic structure occur in the transition-state region, as indicated by Politzer and Toro-Labbe. [6–8] The ΔMEP picture is fully consistent with that based on Δρ, especially for the reactions of the neutral species leading to the neutral products without large charge transfer between the fragments. However, for reactions involving large electron density displacements, the ΔMEP picture is dominated by charge transfer, due to long-range character of electrostatic potential. The analysis of ΔMEP leads to more clear indication of charge shifts than the analysis of deformation density. Thus, it may be concluded that the analysis of the deformation in MEP can be useful as a valuable supplementary tool in combination with the analysis of the deformation density and its components. Certainly, the deformation in MEP reflects changes in all the bonding components (e.g., *s* and *p*), and they are mixed in the overall ΔMEP picture. In the case of deformation density, NOCV method leads to a natural separation of the Δr contributions. For a discussion of separated components of chemical bonds based on deformation in MEP, a decomposition of ΔMEP into the NOCV contributions would be needed. This may be a subject of future research.

## Data Availability

Data is provided within the manuscript or supplementary information files.
